# Lipophilic and Hydrophilic Compounds from *Arthrospira platensis* and Its Effects on Tissue and Blood Cells—An Overview

**DOI:** 10.3390/life12101497

**Published:** 2022-09-26

**Authors:** Friedrich Jung, Steffen Braune, Conrad H. G. Jung, Anne Krüger-Genge, Peter Waldeck, Ingolf Petrick, Jan-Heiner Küpper

**Affiliations:** 1 Institute of Biotechnology, Molecular Cell Biology, Brandenburg University of Technology Cottbus-Senftenberg, 01968 Senftenberg, Germany; 2 Faculty of Health Sciences Brandenburg, Brandenburg University of Technology Cottbus-Senftenberg, 01968 Senftenberg, Germany; 3 Carbon Biotech Social Enterprise AG, 01968 Senftenberg, Germany; 4 Department of Healthcare, Biomaterials and Cosmeceuticals, Fraunhofer-Institute for Applied Polymer Research, 14476 Potsdam-Golm, Germany; 5 Institute of Materials Chemistry, Thermodynamics, Brandenburg University of Technology Cottbus-Senftenberg, 01968 Senftenberg, Germany

**Keywords:** *Arthrospira platensis*, ingredients, nutraceutical, blood cells, tissue cells

## Abstract

The cyanobacterium *Arthrospira platensis* (*Spirulina platensis*) is a natural source of considerable amounts of ingredients that are relevant for nutra- and pharmaceutical uses. Different hydrophilic and hydrophobic substances can be obtained by extraction from the biomass. The respective extraction techniques determine the composition of substances in the extract and thus its biological activity. In this short review, we provide an overview of the hydrophilic compounds (phenols, phycobiliproteins, polysaccharides, and vitamins) and lipophilic ingredients (chlorophylls, vitamins, fatty acids, and glycolipids) of *Arthrospira platensis*. The principal influences of these substances on blood and tissue cells are briefly summarized.

## 1. Introduction

**Figure 1:**
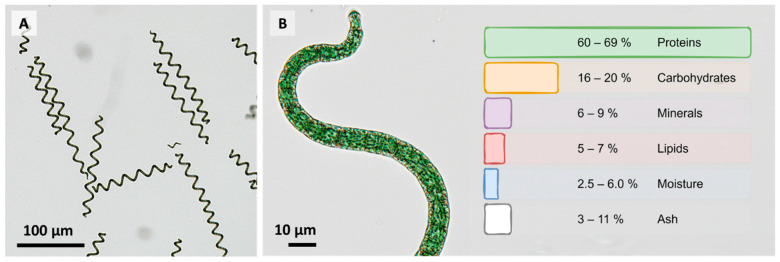
Representative brightfield images of the morphological characteristics of *Arthrospira platensis* (strain: SAG21.99). (**A**) Overview of several sizes (**B**) Detail image of a single spiral and information on the general composition of dried AP biomass [4,5,6,7,8].

Cyanobacteria represent an abundant source of different classes of ingredients of interest for nutraceutical applications [1] or even as pharmaceuticals with potential biological effects on tissue or blood cells [2]. The worldwide interest in such ingredients from microalgae is growing. Especially *Arthrospira platensis* (AP) is one of the richest natural sources of proteins and essential amino acids. The cyanobacterium contains high amounts of proteins, as well as phycocyanin, carotenoids, and essential fatty acids; vitamin B complex and vitamin E; and minerals such as copper, magnesium, iron, selenium, and zinc [3]. Figure 1 shows representative microscopic images of cells of the SAG49.88 strain and summarizes the principal ingredients of AP (Figure 1).

Aside from its majorly nutritionally valuable components, such as carbohydrates, minerals, and proteins (Figure 1 and Table 1), particularly the bioactive compounds extracted from AP have been studied for their therapeutical values. Depending on the extraction process, hydrophilic and lipophilic compounds can be obtained from the AP biomass. Reports about amphiphilic compounds are very sparse. For this reason, they will not be considered further in detail in this review. The type of extraction determines the composition of substances in the extract and, thus, its overall biological activity.

The extraction with water contains proteins such as phycobiliproteins; polyphenols, dimethyl sulfide, and polysaccharides; vitamins B1, B2, B6, and B12; and vitamin C. Moreover, the extraction with ethanol, for example, contains chlorophyll, carotenoids, fatty acids, glycolipids, Pro-vitamin A, vitamin E, vitamin D, and vitamin K.

All of these compounds are described to have different effects on blood or tissue cells. In general, one has to bear in mind that kinds and concentrations of the ingredients depend on the environmental conditions during growth, such as illumination and temperature, nutrients in the culture medium, aeration, and especially the supply with minerals, as well as the conditions during the extraction process [9]. This can explain the broad range of variety of ingredients in different studies. Furthermore, origin and AP strain might play a role here. Despite studies on the biochemical composition of different AP strains are available (e.g., from Aouir et al. [10], Bhattacharya and Shivaprakash [11], and Millia et al. [12]), to our knowledge, a systematic comparison of the concentrations of active substance in the various AP strains with the respective biological effects is not yet available in the literature.

Overall, the different compounds show preventive effects on oxidation, inflammation, and aberrant cell proliferation but can also induce a stimulatory effect on the immune system [13]. In this review, we describe the effects of hydrophilic or lipophilic compounds of AP on blood and tissue cells.

**Table 1:** Minerals in *Arthrospira platensis*, summarized from References [4,5,6,7,8,14].

Component	Amount per 100 g Dry Weight	
Calcium	60–700	mg
Chromium	0.1–0.3	mg
Copper	0.20–1.2	mg
Iodine	0.142–n.a.	mg
Iron	25–100	mg
Manganese	1–5	mg
Magnesium	200–400	mg
Phosphorus	700–1000	mg
Potassium	200–1830	mg
Selenium	0.003–0.010	mg
Sodium	700–1090	mg
Zinc	1–3	mg

## 2. Hydrophilic Compounds

### 2.1. Proteins

Proteins are species-specific; that is, the proteins of one species differ from those of another species. AP is promoted as a valuable source of dietary protein of high nutritional value, containing very high amounts of protein, between 60 and 69 g/100 g dry weight (chicken breast (grilled, without skin), 32 g/100 g; almonds, 21.1 g/100 g), depending on the source [4,5,6,7,8,15]. AP proteins contain all essential amino acids (see Table 2), though with slightly reduced amounts of methionine, cystine, and lysine, as compared to standard proteins, such as those from meat or eggs. It is, however, superior to all standard plant proteins, such as those from legumes. AP has a similar good digestibility of 77.6% as seaweed [16], which is much higher than, for example, soy or legumes. In comparison, hen eggs have an even higher digestibility coefficient of 94.2%. As a result, AP is considered one of the most nutritious foods in the world and was therefore named the “best food for the future” by the United Nations World Food Conference as early as 1974 [17,18].

In conclusion, proteins are of great nutritional value and are involved in the chemical processes essential for the growth of all cells.

### 2.2. Phenols

Microalgae are exposed to ultraviolet light and environmental stressors, which can lead to the formation of free radicals and reactive oxygen species (ROS). Despite their exposure to ROS, cyanobacteria lack oxidative damage in their fatty acids due to protective antioxidant systems comprising vitamins, pigments, and phenols. Here, these compounds are mostly involved in protective activities against, for example, too high illumination to avoid phototoxicity [19]. The polyphenols are divided into phenolic acids, flavonoids, isoflavonoids, stilbenes, lignans, and phenolic polymers [20]. Table 3 summarizes the reported phenols in AP (Table 3).

In comparison to all other phenolic compounds, Phloroglucinol is reported to be the most abundant in AP [21]. This substance is the precursor for polyphenolic compounds with a large chemical variability, the phlorotannins. The latter are majorly found in brown algae and higher plants, for example [22]. Here, they play roles in the protection against UV light and feeding enemies. Gager et al. summarized that—in comparison to these macroalgae—the phenolic content in cyanobacteria is rather low [23]. However, the reports of Goiris and Quéguineur indicate that, in microalgae such as AP, phloroglucinol is majorly acting as an antioxidant [24,25]. According to these reports, the antioxidant activity of phloroglucinol is about 1% to 10% of the overall activity, depending on the respective species. It is recognized that the relatively high degree of hydroxylation (three hydroxyl groups per aromatic core) contributes to the antioxidant activity of phloroglucinol [26]. Beyond that, other bioactivities of phloroglucinol (and derivates) comprise anti-inflammatory, fibrinolytic, and anti-thrombotic, as well as DNA cleaving and enzyme inhibitory, properties [27,28,29].

In humans and animals, the food-derived or supplemented (poly)phenols can act locally and systematically, particularly in the gastrointestinal tract and blood [30].

Substantial research data are available concerning the antioxidant activity of phenols. However, the spectrum of activity is much more complex and comprises, for example, influences on enzyme activity (e.g., angiotensin-converting enzyme inhibition by Apigenin [31]) and protein configuration [32,33,34]. Anti-inflammatory (e.g., suppression of LPS-induced NO synthase-2 and COX-2 activity in mice; downregulation of IL-4 by Apigenin) and antimicrobial (commensal and pathogenic) activities are reported [35,36,37]. The latter can induce changes in the spectrum of the gastrointestinal tract microbiome, as reported, for example, for Catechin [34,38]. Quercetin, for instance, was reported to inhibit DNA topoisomerase and, thus, DNA replication, recombination, and transcription of bacteria [39].

Interestingly, there is also evidence of a mechanism by which (poly)phenols induce oxidative stress, i.e., by generating hydrogen peroxide. In the extracellular environment and blood, hydrogen peroxide can be generated in nM and even µM concentrations and, thus, induce redox cell-signaling pathways or even become cytotoxic [40,41,42]. In cancer cells, the pro-oxidant activity of polyphenols is associated with pro-apoptotic effects [43]. For instance, p-Coumaric acid, was reported to induce apoptosis in human colorectal carcinoma cells.

**Table 3:** Phenolic compounds and superoxide dismutase enzymes in *Arthrospira platensis*, summarized from References [21,44,45].

Component	Amount per 1 g Dry Biomass	
Apigenin	6.00 ± 0.50	ng
p-Coumaric acid	920 ± 90	ng
Catechin	n.a.	
Caffeic acid	n.a.	
Ferulic acid	0.97 ± 0.12	ng
Gallic acid	n.a.	
Genistein	n.a.	
p-Hydroxybenzoic acid	n.a.	
Kaempferol	n.a.	
Naringenin	n.a.	
Naringenin chalcone	n.a.	
Phloroglucinol	51,000 ± 5000	ng
Quercetin	n.a.	
Syringic acid	n.a.	
Vanillic acids	n.a.	
Superoxide dismutase enzymes	392,000	Units

n.a.: not available.

The data available from in vitro and in vivo studies on each of the listed phenols are comprehensive. Displaying all suggested molecular mechanisms is, thus, beyond the scope of this short review. Moreover, the analysis of microalgal-derived (poly)phenolics from aqueous or polar solvent extractions is very complex, especially by the difficulty of separating the individual compounds from the complex mixtures that naturally occur in vivo. There are major differences in the phenolic content of AP that are attributable to both illumination and nutrient levels, thus making a comparison of different studies even more difficult. In addition, phenolic compounds of different microalgae were reported not to be a major contributor to their overall antioxidant capacity [36,46]. It should be noted that most of the evidence comes from in vitro models, and it is unclear if these mechanisms hold true in humans.

### 2.3. Phycobiliproteins

AP contains among many biologically active compounds, phycobiliproteins (phycocyanin, phycoerythrin, and allophycocyanin; see Table 4). Although all of these components exhibit, for example, antioxidant properties, this activity of AP is described to be primarily related to the biliprotein phycocyanin component [47].

Phycobiliproteins are important light-harvesting pigment proteins that are available in cyanobacteria. According to the different composition and absorption spectra, the phycobiliproteins are divided into three categories: phycocyanin (PC, absorption spectrum: 610–640 nm), phycoerythrin (PE, absorption spectra: 500–570 nm), and allophycocyanin (APC, absorption spectra: 650–671 nm).

The content of PC in AP is round about an order of magnitude higher than those of PE or APC. Therefore, in most studies, the effects of PC on cells were investigated. PC was able to scavenge peroxyl, hydroxyl, and peroxyl radicals with a high antioxidant potential [48]. Dartsch reported a dose-dependent inactivation of free superoxide radicals, as well as an anti-inflammatory effect characterized by a dose-dependent reduction of the metabolic activity of functional neutrophils and a dose-dependent inactivation of superoxide radicals generated during an oxidative burst [49]. The anti-inflammatory activity of PC, partly through the inhibition of pro-inflammatory cytokine formation, inducible nitric oxide synthase, and cyclooxygenase-2 expression, has been demonstrated in in vitro, as well as in in vivo, studies [50,51]. In line with these studies, Romay could show that PC inhibited prostaglandin E2 production and also the phospholipase activity [52].

Recent cancer studies revealed a significant dose-dependent inhibitory effect of PC on the growth of cancer cells [2]. Accumulating evidence suggests that PC has a potent anticancer effect on various cancer types (such as breast cancer [53,54], liver cancer [55], lung cancer [56,57], colon cancer [58], leukemia [59], and bone-marrow cancer [60]) in vitro and in vivo. On the other hand, even high-dose PC treatment did not induce significant toxic symptoms or mortality in healthy animals [61,62]. Multiple mechanisms have been found, including the induction of apoptosis, cell-cycle arrest, inhibition of DNA replication, and the generation of ROS [63,64,65,66]. While apoptosis was significantly increased in cancerous cells, PC had considerably lower toxicity on cells from healthy tissues, thus making it an appropriate candidate for chemotherapeutic applications [2,67,68,69].

**Table 4:** Phytopigments in *Arthrospira platensis*, summarized from References [4,5,6,7,8,14,70].

Component	Amount per 100 g Dry Weight	
Chlorophyll-a	1.00–1.70	g
Beta carotene	0.15–0.25	g
Carotenoid (Total)	0.40–0.65	g
Phycocyanin	12.0–19.0	g
Xanthophyll	0.25–0.47	g
Zeaxanthin	0.12–0.20	g

### 2.4. Polysaccharides

Cyanobacterial exopolysaccharides (EPS) are composed of at least 10 different monosaccharides and are characterized by the presence of pentoses, as well as their anionic nature due to the presence of acidic sugars (glucuronic and/or galacturonic acids) and anionic organic (acetyl, pyruvil) and inorganic (phosphate and sulfate) substituents [71]. The carbohydrates typically reported for AP are summarized in Table 5. Sulfated polysaccharides were purified from AP EPS and termed calcium spirulan (Ca-SP). These did not show cytotoxic effects and were reported to be anti-atherogenic and anti-thrombogenic [72]. In addition, sodium spirulan (Na-SP), another sulfated polysaccharide, isolated from AP, was described to exhibit anti-thrombin activity by the activation of heparin cofactor II [73]. In addition, Ca-SP induced the production of tissue-type plasminogen activator t-PA [74].

Sulfated polysaccharides from AP showed anti-coagulant activities; however, they were less than those of heparin [75]. The authors implied that the effect of AP extracts might be due to the presence of uronic acids. The polysaccharides containing uronic acids, due to their negative charge, have the ability for binding calcium ions and therefore might prevent the formation of blood clots.

Ca-SP from AP has also been studied for its antiviral properties [76,77,78,79]. The spirulina polysaccharides inhibited the replication of several enveloped viruses, including herpes simplex virus, influenza virus, measles virus, mumps virus, human cytomegalovirus, and HIV-1 [78,79,80,81]. However, the mechanism of the antiviral activities of these compounds is poorly understood. It is suggested that Ca-Sp selectively interferes at the initial stage of the viral cycle to the host cells [78,79,80].

Furthermore, Ca-Sp appeared to inhibit tumor invasion and metastasis of B16-BL-6 melanoma. This anti-metastasis activity is attributed to blocking the adhesion and migration of tumor cells to laminin substrate and the heparanase activity [82]. The Ca-Sp may inhibit the proliferation of cancer cells by interfering in the synthesis of DNA and RNA [83]. Mittal et al. reported that AP possesses a modulatory effect on hepatic carcinogen metabolizing enzymes that may be involved in antitumor activity [84].

### 2.5. Vitamins (Vitamin C and Vitamins B1, B2, B6, and B12)

Vitamins are essential for health, being precursors of important enzyme cofactors that are required for essential metabolic functions. Table 6 summarizes the vitamins reported in AP. Vitamin C or ascorbic acid is a water-soluble vitamin with antioxidant properties; it is essential for the biosynthesis of many compounds in humans, displaying a great inter- and intra-specific variability [85,86,87]. Vitamin C has been reported as a regulator of Hypoxia-Inducible Factor 1α [88,89], a major microenvironmental driver of carcinogenesis and tumor angiogenesis. Vitamin C also has effects on extracellular matrix (ECM), impaction on collagen biosynthesis, and deposition [90,91].

It seems to be uncertain whether AP contains vitamin C. The dependence of the concentration seems to be particularly strong on the influencing factors since vitamin C could be detected in some studies while not in others [92].

While only about 7% of the total population suffers from a vitamin B12 deficiency, among vegans, it is 60 to 90%, depending on the study. Therefore, AP is often used as a vegan source of vitamin B12, a water-soluble vitamin that is present in meat products but absent in plants. High levels of vitamin B12 are described in the nutritional labels of dietary supplements that contain edible cyanobacteria, such as *Spirulina*, *Aphanizomenon*, and *Nostoc* [93]. However, although substantial amounts of vitamin B12 were detected in these commercially available supplements, using a microbiological vitamin B12 assay method, these supplements often contained large amounts of pseudovitamin B12 (Coα-[α-(7-adenyl)]-Coβ-cyanocobamide) [94,95,96,97,98,99], which is biologically inactive in humans, and only 17% were identified as dimethylbenzimidalylcobamide, also known as vitamin B12 [98,100]. Therefore, edible cyanobacteria and their products are only of limited use as sources of vitamin B12 for vegetarians; see the statement of the *American Dietetic Association* [100].

## 3. Lipophilic Compounds

### 3.1. Chlorophylls

Chlorophyll-a is the lipid-soluble pigment of chlorophylls, the primary photosynthetic pigment in all algae, and the only chlorophyll of cyanobacteria. The total amount of chlorophyll in algae is in the range of 0.5 to 1.5% of dry weight [101]. Chlorophyll or its derived products are known for their health benefits, due to their antioxidant and apoptotic properties. Chlorophyll and other tetrapyrrolic compounds, which are structurally related to bilirubin (the potent antioxidant bile pigment) [102], are among the important candidate molecules, which are considered to be responsible for this protective effect [103,104].

Chlorophyll displayed an antioxidant activity when administered orally to Wistar rats (for 14 days at 8 and 16 µg/mL) injured with the pro-oxidant sodium nitrate compound, revealing a strong in vivo antioxidant activity of chlorophylls [105].

Chlorophylls have been shown to produce anti-proliferative effects in pancreatic cancer cell lines (PaTu-8902, MiaPaCa-2, and BxPC-3) in a dose-dependent manner (10–125 μmol/L) [106]. Importantly, chlorophyll-mediated suppression of pancreatic cancer cell viability has been replicated in in vivo experiments, where the administration of chlorophyll-a resulted in the significant reduction of pancreatic tumor size in xenotransplanted nude mice [106]. In trouts, chlorophyll was able to avoid dibenzo[def,p]chrysene (DBC)-induced DNA adduct formation when it was used as a diet (4000 ppm) [107].

### 3.2. Vitamins (Vitamin D, Vitamin E, Tocopherols and Tocotrienols, Vitamin K, and Provitamin A)

Vitamin D exists in five forms: D1 to D5. The main forms of vitamin D in humans are D2 and D3. Numerous studies have reported on the health benefits of vitamin D in cancer prevention and anti-neurodegenerative effects [108,109,110,111,112,113]. Although poorly documented, it is known that microalgae can contain vitamins D2 and/or D3 [114,115]. Vitamin D has been reported to exert chemoprevention activities through antiproliferative and immune modulatory effects on tumor cells in vitro. In addition, vitamin D diminishes the growth of cancer cells in vivo [108] by blocking cell-cycle progression due to (i) increasing the expression of cyclin-dependent protein-kinase-inhibitors p21 and p27 [116,117], (ii) modulating the expression of insulin growth factor (IGF-1) [118,119], (iii) blocking cell proliferation via Wnt/β-catenin-signaling pathways [120,121], and (iv) inducing apoptosis or autophagy [122].

Vitamin E or tocopherol is synthesized in many microalgae—including AP—and, thus, can be a valuable source of this vitamin. It has been reported that the tocopherol content is comparable to or higher in microalgae than in edible terrestrial plants [123,124]. Tocopherols and tocotrienols are liposoluble antioxidants, protecting membrane lipids from oxidative damage, since they are chain-breaking molecules that are able to prevent the propagation of lipid peroxidation. Vitamin E blocks the production of ROS and lipid peroxidation and is involved in the inhibition of low-density lipoprotein oxidation, a process known to have a role in the development of atherosclerosis [125,126,127,128]. Vitamin E can have a chemoprotective role, reducing the risk of pancreatic cancer in mice (80% tumor growth inhibition at 100 mg/kg) [129,130]. The Phosphoinositide 3-Kinase pathway is involved in the activity of vitamin E and the inhibition of prostate-cancer cell growth [131]. Vitamin E improves endothelial function and vascular health and reduces vascular damage [132,133].

Microalgae can contain vitamin K in concentrations between 6.5, and 12.7 μg per g dry weight and AP up to 1.05 mg per 100 g dry weight (see Table 6) [134]. Vitamin K is a key regulator for the synthesis of blood-clotting factors in the liver. It is associated with disorders mainly related to coagulation. In particular, vitamin K deficiency is also linked to other pathological conditions, such as malabsorption disorders, antibiotics, and drug interactions, especially with coumarin-based anticoagulants [135,136].

Provitamin A (β-carotene) is a naturally occurring vitamin A precursor, which is a strongly colored red-orange pigment and the most active and important provitamin A carotenoid. Provitamin A has been reported to have antioxidant and anti-inflammatory activities [137,138,139] protecting against singlet oxygen-mediated lipid peroxidation [139]. Moreover, β-carotene inhibited the production of nitric oxide and prostaglandin E2 and suppressed the expression of iNOS, COX-2, TNF-α, and IL-1β. The suppression of such inflammatory mediators by β-carotene is discussed to result from its inhibition of NF-κB activation through blocking nuclear translocation of the NF-κB p65 subunit [138]. In addition, β-carotene suppressed the transcription of inflammatory cytokines, including IL-1β, IL-6, and IL-12, in a macrophage cell line stimulated by lipopolysaccharide (LPS from Gram-negative bacteria) or IFNγ [137].

Beyond the anti-inflammatory effects, there seems to be a slight but significant inverse correlation between the intake of β-carotene and the development of prostate cancer [140]. Supplemental beta-carotene intake at a dose level of at least 2 mg per day was associated with decreased prostate-cancer risk in men with low (below the median of 4129 µg per day) dietary beta-carotene intake (RR = 0.52; 95% CI = 0.33 to 0.81). Among men with low dietary beta-carotene intake, the age-adjusted rate of prostate cancer was 1122 per 100,000 person-years in those who did not take supplemental beta-carotene and 623 per 100,000 person-years in those who took at least 2 mg per day of supplemental beta-carotene. These results do not provide strong support for the population-wide implementation of high-dose antioxidant supplementation for the prevention of prostate cancer. However, beta-carotene supplementation in men with low dietary beta-carotene intakes was associated with a reduced risk of prostate cancer. One must keep in mind that the dosages used in these studies are much higher than the daily intake recommended by the World Health Organization, with gram quantities consumed daily for months [141,142].

### 3.3. Lipids and Fatty Acids

Microalgal lipids have gained significant importance—besides their possible suitability as feedstock for biofuels production—as important biological molecules for the treatment of inflammatory pathologies [143]. According to a study by Ramadan and coworkers, AP contains about 45% neutral lipids, 39% glycolipids, and 16% phospholipids (all values refer to the amount of total lipids) [144]. Table 7 summarizes the lipid sub-classes reported in the literature [144,145]. The most abundant lipids are triacylglycerols (neutral lipids), with 243 ± 3.16 g/kg of total lipids, and sulphoquinovosyl diacylglycerol (SQDG, glycolipids), with 198 ± 3.50 g/kg of total lipids [144]. Sterol esters (neutral lipids) and digalactosyl diacylglycerol (DGDG, glycolipids) account for about 90.7 ± 2.10 g/kg and 83.5 ± 2.55 g/kg of total lipids, respectively. All other lipids exhibit quantities below 64 g/kg of total lipids and can be reviewed in the abovementioned manuscript [144].

Particularly glycolipids—which are mainly located in the thylakoid membranes of AP—show interesting bioactive effects [145]. Their principle structure consists of a carbohydrate moiety that is beta-monogalactosyl diacylglycerol (MGDG) and (DGDG) or alpha-linked sulfoquinovosylacyl glycerol (SQAG) to the sn-3 position of glycerol, which is acylated at the residual hydroxyls by fatty acids of different lengths and degrees of unsaturation [146].

One study by Chirasuwan et al. reported an anti-Herpes Simplex Virus type 1 (HSV-1) activity of AP-derived SQDG in kidney fibroblasts (African green monkey-derived Vero cells, IC50 = 6.8 µg/mL) [147]. A relatively early report by Ayehunie et al. revealed that AP-extracts show activities against the human immunodeficiency virus (HIV-1), as well [148]. However, potentially responsible substances were not identified in this study. Despite AP-derived sulfated polysaccharides (calcium spirulan) being also reported to have antiviral properties (see Section 2.4), it is worth noting that Gustafson and Reshef reported an anti-HIV-1 activity of SQDG and diacylated sulfoglycolipids, as well as acylated diglycolipids [149,150]. However, in these studies, the substances were extracted from other cyanobacteria, such as *Lyngbya lagerheimii*, *Phormidium tenue*, *Oscillatoria raoi*, *O. trichoides*, and *O. limnetica*.

Other glycolipids present in AP, such as MGDG and DGDG, showed anti-inflammatory effects in vivo. Particularly the first was more efficient than the reference drug indomethacin in a carrageenan-induced mouse-paw oedema model [151].

A study with the microalga *Pavlova lutheri* revealed that eicosapentaenoic acid (EPA) was especially concentrated in MGDG (45%), as well as that docosahexaenoic acid was dispersed within triacylglycerol (27%), diacylphosphoglycerol (22%), and betaine lipids (21%) [152]. All of these lipids could have an important role in inflammatory diseases. Methanolic extracts showed no inhibitory activity on (Gram-negative bacteria) LPS-induced NO production in RAW264.7 macrophage cells [153]. A similar extract inhibited LPS-induced NO production in the same cell line through the downregulation of iNOS [154]. In the same line, DGDG and SQDG from the Brown alga *Sargassum horneri* caused an induction of apoptosis through DNA fragmentation in Caco-2 colon cells [155]. Thus, the bioactive effects of glycolipids in inflammatory processes appear to involve NO, but more studies are necessary to investigate this role in the respective pathologies.

Phytosterols represent a class of interesting amphiphilic compounds. Moreover, for these substances, only a few reports about the identification and bioactivity of AP-derived sterols other than cholesterol (<0.1 mg per 100 g dry weight) are available. Two studies indicate beta-sitosterol and stigmasterol in the non-saponifiable lipid fraction of AP extracts (>10% each of this fraction) [156,157]. In *A. maxima*, D^7^-avena sterol, campesterol, and ergosterol have also been identified [158].

Among the lipids, glycolipids are particularly quite abundant in microalgae and are considered an important source of fatty acids. The fatty acids reported in AP are summarized in Table 8. The most studied for the pharmacological potential of these compounds are polyunsaturated fatty acids.

Linoleic acid and gamma-linolenic acid belong to this group and account for up to about 17% and 21% of the total fatty acid content of AP, respectively. The influence of both on human health was studied in several clinical trials. For instance, in a Spanish multicenter matched case-control study (EpiGEICAM), the serum levels of phospholipids fatty acids were associated with breast-cancer subtypes. Their data showed that women with high levels of linoleic acid and arachidonic to dihomo-γ-linolenic acid ratio had lower risks for breast cancer [159]. A meta-analysis by Zhou et al. concerning the relationship between serum and dietary linoleic acid and breast-cancer risk came to the same conclusion [160]. The intake of linoleic acid can result in a reduced risk for breast cancer. The molecular mechanisms behind this are yet not fully understood. Interestingly, a recent study by Ogata et al. revealed that long-term treatment of mouse colorectal cancer cells (CT26 cell line) with linoleic acid induced a quiescent cell phenotype. The cells remained dormant after subcutaneous inoculation into a syngeneic mouse model, which was fed with linoleic acid subsequently. On a molecular level, glycolysis and oxidative phosphorylation, as well as the expression of the regulatory factors MycC and Pgc1α, were reduced in the CT26 cells [161]. The profiling of micro-RNA expression revealed an upregulation of miR-494, which was concluded to be majorly involved in linoleic acid–induced dormancy in cancer cells.

A similar conclusion was drawn by Ohmori and colleagues, who studied the influence of linoleic acid on two human gastric cancer cell lines (MKN28 and MKN45) [162]. Their results showed reduced tumor growth when cells were treated with linoleic acid before implantation in a nude mouse model. Levels of VEGF, EGFR, and Bak were decreased, and Bcl-2 levels were increased in the cells, emphasizing that linoleic acid contributes to the induction of quiescence and subsequent dormancy in cancer cells.

Likewise, another recent meta-analysis of prospective cohort studies emphasized the positive effect of a long-term intake of polyunsaturated fatty acids—particularly linoleic acid—on the risk for cancer but also for cardiovascular disease [163]. Farvid and coworkers reviewed that dietary intake of linoleic acid reduces the risk of coronary-heart-disease events by about 15% and the risk for deaths caused by coronary heart disease by about 21% [164]. Altogether, the consumption of polyunsaturated fatty acids could have beneficial effects in the resolution of inflammatory processes and, thus, prevent their progression to cancer.

Palmitic acid is a saturated fatty acid that accounts for between 18% and 46% of the total fatty acids in AP (see Table 8). Beyond its uptake as a dietary fatty acid, it is also synthesized in the human body through de novo lipogenesis [165]. It is the most common saturated fatty acid in the human body (20–30% of total fatty acids), a source of energy, and essential for lipid metabolism. It is a component of cell membranes and is required for palmitoylation of proteins and respective signaling molecules and for maintaining an efficient lung surfactant activity [166]. In principle, the roles of palmitic acid as an intracellular signaling molecule are diverse. The underlying molecular mechanisms concerning the more beneficial or pathological roles are still the subject of research in different areas.

Several reports indicate an involvement of palmitic acid in disease developments such as metabolic syndrome, cancer, cardiovascular and neurodegenerative diseases. A substantial review of the available study data and proposed molecular mechanisms was published by Fatima and coworkers in 2019 [167].

Clinical data indicate that type 2 diabetes and metabolic syndrome are associated with high dietary consumption and associated elevated levels of palmitic acid in human blood plasma (e.g., diabetes: three-fold higher compared to normal) [168,169,170]. The latter results in an increased uptake of the fatty acid into the cells and in an upregulation of the respective—non-oxidative—metabolic pathways (e.g., diacylglycerol, protein kinase C, and insulin receptor substrate-1) [171]. Taken together, this can inhibit insulin signaling and cause insulin resistance [172].

Reports on the influence of palmitic acid on the cardiovascular system similarly indicate its diverse roles. On the one hand, normal plasma levels (about 150 µM) are rather protective, e.g., through activation of 5′ AMP-activated protein kinase, glucose transporter 4 expression, and PCKζ phosphorylation [173,174,175]. Reports indicate positive influences on cardiomyocyte viability and function and a protective role against myocardial infarction [176]. On the other hand, increased levels can promote the development of cardiovascular diseases. Among others, this comprises dysfunction of cardiomyocytes (e.g., caveolin-3 loss and inhibited Ca^2+^ release) and endothelial cells (e.g., Hippo-Yes-associated protein phosphorylation and increased Ste20-like kinase 1) [177,178,179]. Moreover, inflammation of the endothelium (e.g., induction of interferon regulatory factor-3) and impaired angiogenesis are reported, i.e., in the form of inhibited progenitor cell proliferation and migration (e.g., through inhibited STAT5 transcription) [177,180,181]. Based on these data, guidelines for the prevention of cardiovascular diseases recommend reducing excessive consumption of saturated fatty acids [182,183].

Furthermore, concerning cancer development, reports about the role of dietary palmitic acid and the involved molecular pathways are controversial. Similar to linoleic acid, it was shown to inhibit proliferation and metastasis of prostate cancer cells in a nude mouse xenograft model. In this study, Zhu et al. identified suppression of the PI3K/Akt pathway by palmitic acid as one underlying molecular mechanism [184]. The induced cell cycle arrest (G1 phase) was associated with an increased expression of p27 and a decreased expression of p-RB and cyclin D1. The data further indicated that p-Integrinβ1 and PKCζ suppression and an increased E-cadherin expression are involved in reduced cell metastasis. A study by Wu and colleagues proposed the potential of palmitic acid (resp. palmitate) as an adjuvant in endometrial cancer therapy [185]. Their results revealed increased chemosensitivity of HEC-1-A and RL95-2 cells when doxorubicin or cisplatin treatments were supplemented accordingly. The cells furthermore showed increased levels of DNA damage, autophagy, and apoptosis, as well as cell-cycle arrest.

Concerning cell cycle arrest/delay and apoptosis, similar data were reported for HER2/neu-positive breast cancer cells [186]. In this study, the endoplasmic reticulum stress response was partially activated by exogenous palmitate. This resulted in a reduced HER2 and HER3 expression and increased sensitivity for trastuzumab, a humanized therapeutic monoclonal antibody against HER2 receptor-positive cancer cells.

In contrast with these findings, another in vivo study in mice revealed that oral and skin cancer cells or tumors which were exposed to palmitic acid in vitro remained metastatic after implantation [187]. The cells kept the strongly metastatic phenotype without further supplementation of the palmitic acid in the animals. This phenomenon was termed “prometastatic memory”. Earlier data from this group indicated that a high-fat diet upregulated the metastatic potential of CD36-positive metastasis-initiating cells in immunodeficient/immunocompetent orthotopic mouse models of human oral cancer. An antibody-based blocking of the CD36 receptor inhibited metastasis [188]. It should be noted that the EpiGEICAM study also revealed that participants with high serum concentrations of palmitoleic acid had higher risks for breast cancer. This monounsaturated fatty acid accounts for up to 1.5% of the total fatty acid content of AP. Fatima et al. summarized the studies indicating that high plasma levels of saturated fatty acids also play a role in neurodegenerative diseases and inflammation [167].

In summary, there are increasing data available concerning the roles of particularly palmitic acid as a pathophysiological signaling molecule beyond its other physiological functions mentioned above. However, these principle findings are very much related to the frequent consumption of foods that—in comparison to AP (up to 2.5 g/100 g; see Table 7)—contain very high levels of this saturated fatty acid, such as palm oil (ca. 41 g/100 g), butter (ca. 26 g/100 g), and lard (ca. 23 g/100 g), as well as fatty meat, e.g., loin from pork (ca. 20 g/100 g) (https://wholefoodcatalog.info/nutrient/palmitic_acid/foods/high/, accessed on 1 April 2022).

Other fatty acids, such as myristic and oleic acid, are also reported to have beneficial effects on human health, e.g., the gut microbiota, cancer, obesity-related disorders, and cardiovascular disease [189,190,191,192,193,194]. Although the available amounts in AP are relatively low (below 5% of total fatty acids), a cumulative effect might be conceivable [195].

## 4. Conclusions

**Figure 2:**
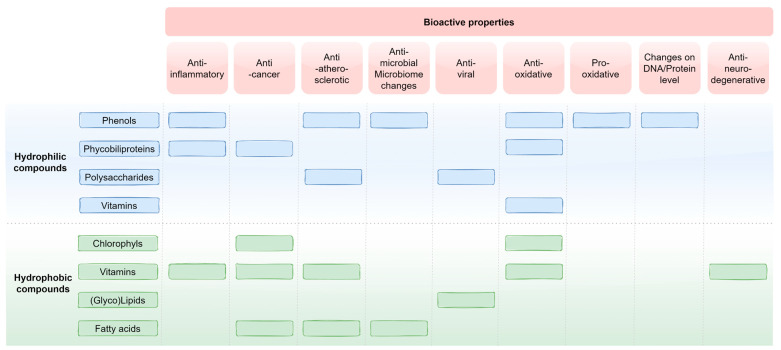
Overview of hydrophilic and hydrophobic compounds and their respective bioactive properties.

AP cyanobacteria contain a variety of biologically active hydrophilic and lipophilic compounds, which are reported to have therapeutic effects on tissue, as well as blood cells, in many studies (Figure 2). Despite the fact that each substance can act solely and concentration-dependent, different compounds can work synergistically, as well. Such an interplay of a few or many substances can enhance the biological effects. For example, antioxidant-active compounds are contained in both the water-soluble (phycocyanin, chlorophyll, and superoxide dismutase) and the fat-soluble extract (beta-carotene and w-6 fatty acids). Thus, we can assume that the complete AP powder has stronger antioxidative effects than the two isolated extracts alone. This exact synergistic effect could already be shown in a first study. Based on the DPPH assay [196], the radical scavenging activity of the aqueous extract was higher than that of isolated phycocyanin [197].

However, to achieve a broad range of protective effects, a synergy between groups of these ingredients is likely to be beneficial. The combination of the two extracts or the use of whole AP powder might result in enhanced preventive activities’ action by blocking (i) reactive oxygen/nitrogen species generation, (ii) inflammation, and (iii) aberrant cell proliferation (such as cancer cells) and (iv) by stimulation of the immune system.

However, it must be pointed out that the concentrations of the different ingredients in AP can vary greatly. For example, in the case of nitrate deficiency in the culture medium, the content of certain polyunsaturated fatty acids can be halved [198] Furthermore, the concentration of phycocyanin strongly depends on the illumination [199], and the polysaccharide concentration is elevated under stress conditions [200]. Thus, it is difficult to predict the effect of extracts but also of AP powders or tablets without appropriate pre-analyses.

## Figures and Tables

**Table 2:** Amino acids in *Arthrospira platensis*, summarized from References [4,5,6,7,8,14].

Component Class	Component	Amount per 100 g Dry Weight	
Nonessential amino acids	Alanine	4.0–5.0	g
	Arginine	3.0–5.0	g
	Aspartic acid	1.5–5.9	g
	Cystine	0.5–0.75	g
	Glutamic acid	6.0–9.1	g
	Glycine	2.0–4.0	g
	Proline	2.0–3.0	g
	Serine	2.7–4.5	g
	Tyrosine	1.0–3.0	g
Essential amino acids	Histidine	0.5–1.5	g
	Isoleucine	3.0–4.0	g
	Leucine	3.0–6.0	g
	Lysine	2.9–6.0	g
	Methionine	1.0–6.0	g
	Phenylalanine	2.5–3.5	g
	Threonine	1.5–3.0	g
	Tryptophan	0.9–2.0	g
	Valine	1.0–3.5	g
Non-proteinogenic amino acids	Theanine	2.97–n.a.	g

**Table 5:** Carbohydrates in *Arthrospira platensis*, summarized from References [4,5,6,7,8,14].

Component	Amount per 100 G Dry Weight	
Galactose	3	g
Glucose	54.4	g
Mannose	9.3	g
Rhamnose	22.3	g
Xylose	7	g

**Table 6:** Vitamins in *Arthrospira platensis*, summarized from References [4,5,6,7,8,14].

Component		Amount per 100 g Dry Weight	
Biotin		0.005–n.a.	mg
Folic acid		0.05–0.30	mg
Inositol		70–90	mg
Vitamin A *	(Provitamin A as beta-Carotene)	150–250	mg
Vitamin B1	(Thiamine)	1.5–4.0	mg
Vitamin B2	(Riboflavin)	3–5	mg
Vitamin B3	(Niacin)	10–25	mg
Vitamin B6	(Pyridoxine)	0.5–0.7	mg
Vitamin B12	(Cobalamin)	0.05–2.0	mg
Vitamin E	(Tocopherol)	5–20	mg
Vitamin K		0.90–1.05	mg

* Vitamin A (as beta-Carotene): 352,000 IU [7].

**Table 7:** Lipids in *Arthrospira platensis*, summarized from References [144,145].

Neutral Lipids	Glycolipids	Phospholipids
Free fatty acids	Cerebrosides	Phosphatidylcholine
Free sterols	Digalactosyl diacylglycerol	Phosphatidylethanolamine
Diacylglycerols	Esterified steryl glucoside	Phosphatidylinositol
Monoacylglycerols	Monogalactosyl diacylglycerol	Phosphatidylserine
Sterol esters	Steryl glucoside	
Triacylglycerols	Sulphoquinovosyl diacylglycerol	

**Table 8:** Fatty acids in *Arthrospira platensis*, summarized from References [4,5,6,7,8,14].

Component Class	Component	Amount per 100 g Dry Weight	
Saturated fatty acids (1.95 g per 100 g dry weight)	in total	33.68–66.75	% ^1^
	Pentadecenoic	1.26–3.16	% ^1^
	Pentadecanoic acid	0.70–1.53	% ^1^
	Caprylic acid	3.65–3.73	% ^1^
	Palmitic acid	18.00–46.07	% ^1^
	Stearic acid	0.95–1.41	% ^1^
Polyunsaturated fatty acids (1.93 g per 100 g dry weight)	in total	28.2–47.8	% ^1^
	Linoleic acid	16.18–17.43	% ^1^
	γ-Linolenic acid	8.87–21.73	% ^1^
	Hexadecadienoic acid	2.43–3.38	% ^1^
Monounsaturated fatty acids (0.26 g per 100 g dry weight)	in total	n.a.	
	Palmitoleic	1.00–1.50	% ^1^
	Oleic acid	1.97–5.23	% ^1^
Trans fatty acids	in total	0.3–0.5	% ^1^
Cholesterol	in total	<0.1	mg

^1^ Percent of total fatty acids.

## Data Availability

All data in this review article are summarized from previously published papers.
